# Supplementary material for: Major Complex Trait for Early De Novo Programming ‘CoV-MAC-TED’ Detected in Human Nasal Epithelial Cells Infected by Two SARS-CoV-2 Variants Is Promising to Help in Designing Therapeutic Strategies
Source: Vaccines (Basel). 2021 Nov 26;9(12):1399. doi: 10.3390/vaccines9121399 (PMC8708361; doi:10.3390/vaccines9121399)
Supplement: Supplementary file 1 [file vaccines-09-01399-s001.zip › vaccines-1402544-supplementary.pdf]

Figure S1: Transcript accumulation of selected *ReprogVirus* marker genes in human nasal epithelial cells infected with two SARS-CoV-2 variants at 8 hours post infection (hpi), 24 hpi and 72 hpi. Transcript levels are averages from three cell origins (donators/cell cultures) given in % of 0 hpi. A – SARS-CoV-2 (virus originally discovered); B – SARS-CoV-2 Δ382. Different letters indicate significant differences between net RPKM for α = 0.05. Letters on the 100% horizontal line correspond to 0 hpi.

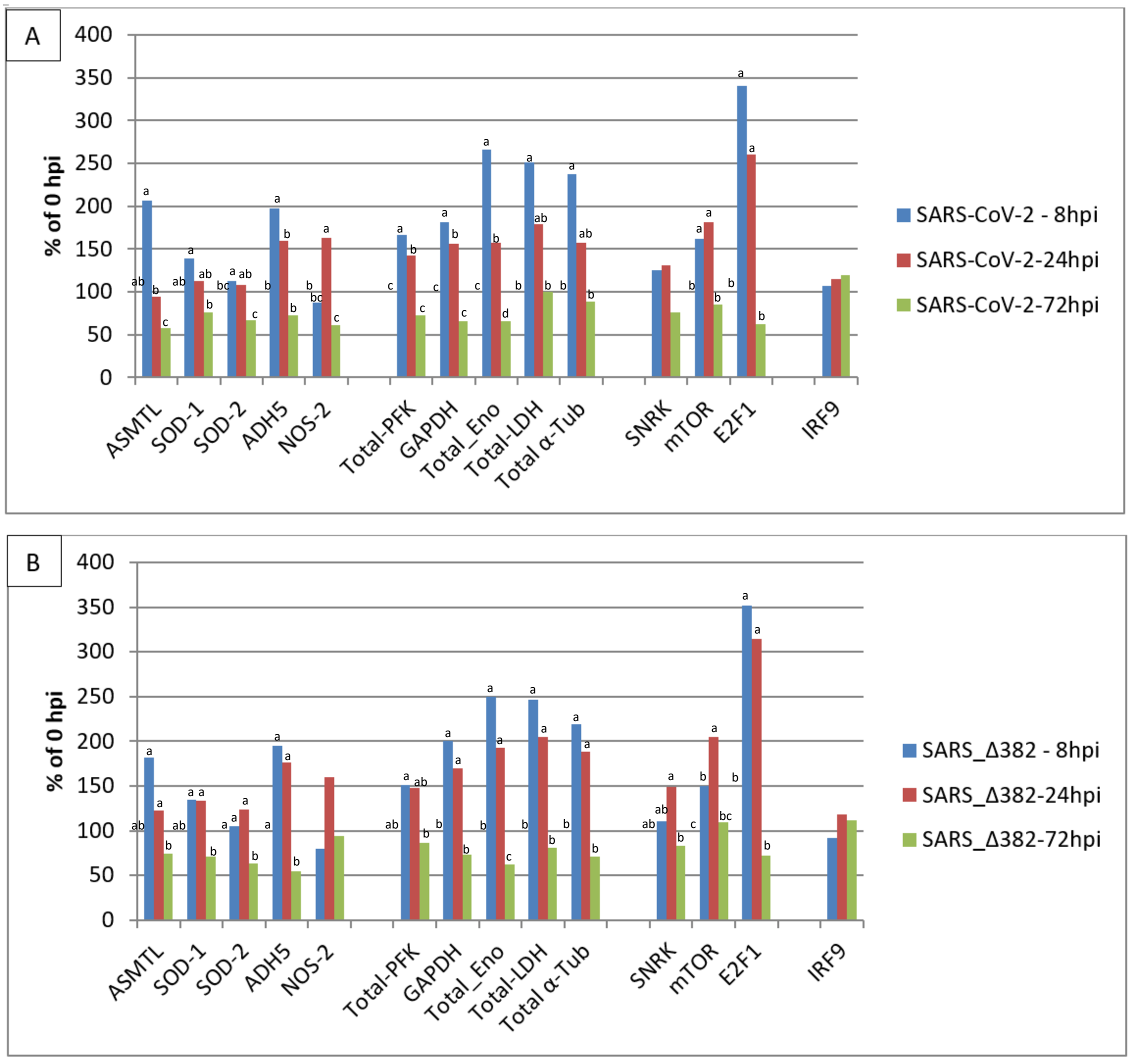

Table S1: RPKM mean values ± SE corresponding to virus infection in nasal cell on the Figure 1. A) original virus SARS-CoV-2, and B) SARS-CoV-2 mutant Δ382. Number of biological replicates: 3. ASMTL (*Acetylserotonin O-Methyltransferase Like*), *SOD1* (*Superoxide dismutase 1*), *SOD2* (*Superoxide dismutase 2*), *ADH5* (*Alcohol dehydrogenase 5*), NOS-2 (*Nitric oxide synthase-2*), Total PFK (*Phosphofructokinase PFKM + PFKL + PFKP*), GAPDH (*Gyceraldehyde-3-phosphate dehydrogenase*), Total Eno (*Enolase 1 + 2 + 3*) Total *LDH (Lactate dehydrogenase A + B + C + AL6A + AL6B)*, alpha Tub (alpha tubulin *TUB-A1B* + *TUB-A1C* + *TUB-A4A*), SNRK (*Sucrose nonfermenting (SNF)-related kinase*), mTOR (*mammalian target of rapamycin*), E2F1 (*E2F transcription factor 1*), IRF9 (*Interferon regulatory factor 9*).

**Treatment (virus/time of infection)**		**ASMTL**	**SOD-1**	**SOD-2**	**ADH5**	**NOS-2**	**Total PFK**	**GAPDH**	**Total Eno**	**Total LDH**	**Total alpha-Tub**	**SNRK**	**mTOR**	**E2F1**	**IRF9**
**original virus SARS-CoV-2**	**0h**	2.82±0.22	294.29 ± 39.42	43.72 ± 1.25	30.43 ± 1.74	12.06± 0.32	47.66 ±4.67	599.5±70.34	139.68 ± 7.71	210.06 ± 17.09	506.03 ± 43.36	4.73 ± 0.81	5.31 ± 0.2	0.82 ± 0.08	20.26 ± 0.51
	**8h**	5.85±0.7	409.46 ± 4.37	42.93 ± 2.77	60.01 ± 5.58	10.62± 0.36	79.19 ±5.5	1087.84 ±23.69	372.19 ± 28.27	526.98 ± 47.34	1203.65 ± 52.88	5.94 ± 0.18	8.56 ± 1.07	2.8 ± 0.33	21.63 ± 0.18
	**24h**	2.65±0.28	330.33 ± 11.75	41.26 ± 3.21	48.52 ± 7.06	19.67± 1.28	67.93 ±5.36	938.12± 19.58	219.87 ± 17.09	375.7 ± 32.86	793.88 ± 29.92	6.2 ± 0.08	9.64 ± 0.35	2.14 ± 0.09	23.23 ± 1.26
	**72h**	1.61±0.27	224.89 ± 11.49	25.68 ± 2.93	22.26 ± 0.47	7.39±1.18	34.48 ±4.02	397.4±38.1	92.58 ± 1.42	209.29 ± 4.09	447.02 ± 88.7	3.58 ± 0.82	4.54 ± 0.64	0.51 ± 0.07	24.3 ± 2.97
**SARS-CoV-2 mutant Δ382**	**0h**	2.82±0.22	294.29 ± 39.42	43.72 ± 1.25	30.43 ± 1.74	12.06± 0.32	47.66 ±4.67	599.5±70.34	139.68 ± 7.71	210.06 ± 17.09	506.03 ± 43.36	4.73 ± 0.81	5.31 ± 0.2	0.82 ± 0.08	20.26 ± 0.51
	**8h**	5.14±0.72	394.46 ± 20.58	39.82 ± 3.27	59.34 ± 7.55	9.58±1.85	72.22 ±3.49	1200.26 ±36.14	348.54 ± 22.46	517.72 ± 38.32	1110.91 ± 92.61	5.25 ± 0.54	7.96 ± 0.35	2.89 ± 0.5	18.52 ± 1.86
	**24h**	3.47±0.21	391.85 ± 16.76	46.97 ± 2.16	53.75 ± 1.68	19.29± 2.75	70.41 ±4.66	1014.64 ±32.38	269.85 ± 26.25	429.94 ± 25.5	954.01 ± 36.91	7.03 ± 0.18	10.87 ± 0.27	2.59 ± 0.33	23.85 ± 0.91
	**72h**	2,1±0,37	209,78± 1,51	23,96± 1,25	16,66 ±1,93	11,33± 0,07	41,29 ±3,67	435,29± 36,6	87,08± 2,99	170,64± 7,31	360,19± 11,66	3,91±0,8	5,79±0,84	0,59±0,04	22,59± 1,79
